# Quadratic Meta-Reflectors Made of HfO_2_ Nanopillars with a Large Field of View at Infrared Wavelengths

**DOI:** 10.3390/nano10061148

**Published:** 2020-06-11

**Authors:** Feng Tang, Xin Ye, Qingzhi Li, Hailiang Li, Haichao Yu, Weidong Wu, Bo Li, Wanguo Zheng

**Affiliations:** 1 Research Center of Laser Fusion, China Academy of Engineering Physics, Mianyang 621900, Sichuan, China; tangfengf3@126.com (F.T.); dearlqz@163.com (Q.L.); wuweidongding@163.com (W.W.); Lb6711@126.com (B.L.); 2 Key Laboratory of Microelectronic Devices & Integrated Technology, Institute of Microelectronics of Chinese Academy of Sciences, Beijing 100029, China; lihailiang@ime.ac.cn; 3 Suzhou Institute of Nano-Tech and Nano-Bionics, Chinese Academy of Sciences, Suzhou 215125, Jiangsu, China; hc.yu@hotmail.com; 4 IFSA Collaborative Innovation Center, Shanghai Jiao Tong University, Shanghai 200240, China

**Keywords:** metasurface, quadratic reflector, a large field of view, refractory material

## Abstract

Metasurfaces, being composed of subwavelength nanostructures, can achieve peculiar optical manipulations of phase, amplitude, etc. A large field of view (FOV) is always one of the most desirable characteristics of optical systems. In this study, metasurface-based quadratic reflectors (i.e., meta-reflectors) made of HfO_2_ nanopillars are investigated to realize a large FOV at infrared wavelengths. First, the geometrical dependence of HfO_2_ nanopillars’ phase difference is analyzed to show the general principles of designing infrared HfO_2_ metasurfaces. Then, two meta-reflectors with a quadratic phase profile are investigated to show their large FOV, subwavelength resolution, and long focal depth. Furthermore, the two quadratic reflectors also show a large FOV when deflecting a laser beam with a deflecting-angle range of approximately ±80°. This study presents a flat optical metamaterial with a large FOV for imaging and deflecting, which can greatly simplify the optical–mechanical complexity of infrared systems, particularly with potential applications in high-power optical systems.

## 1. Introduction

In recent years, 2D metamaterials, i.e., metasurfaces, have emerged as a promising alternative to traditional bulky optical components [1,2,3,4], achieving miniature optical systems with many peculiar properties. Metasurfaces, being composed of subwavelength nanostructures, can compress the thickness of optical elements to the subwavelength level and realize an arbitrary manipulation of phase, amplitude, etc. With artificially designed electromagnetic properties, metasurfaces have great potentials in the applications of sensing [5,6], solar cells [7,8], holographic [9,10], etc. Except for far-field modulation such as artificial focus patterns [11], metasurfaces could be exploited for near-field functionalities [12]. Now, the metasurfaces develop in many fields, even with artificial intelligence [13]. In particular, the all-dielectric metasurfaces have attracted much interest of researchers [3,14,15,16,17], due to low loss, high efficiency, low cost, and CMOS-compatibility. The all-dielectric feature introduces many application potentials via replacing conventional optical systems, such as ultra-compact structured light projectors [18]. Researchers have demonstrated an array of high-performance all-dielectric metasurfaces, paving the way for metamaterials towards multi-functional compacted optical systems.

A large field of view (FOV) is always one of the most desirable characteristics for optical systems [19], such as imaging [20,21,22], beam-deflecting [23,24], solar power [25,26], etc. However, the conventional configurations of wide-angle-view optical systems are always achieved via a 3D configuration, such as Luneburg lens [27] and compound eyes [20,28], leading to bulky and expensive systems. Recently, some works are reported on the metalens with large FOVs in the visible near-infrared ranges based on compact nanoscatters [29] and asymmetric nanodimers [30]. Moreover, a metallic metasurface with a quadratic phase profile realized a wide range of operation angles [31]. However, these works are based on non-refractory materials for the non-high-power systems. For the applications in the high-power systems, the refractory materials should be employed to construct metasurfaces. The refractory ceramic material, hafnium oxide (HfO_2_), is usually employed in high-power optical components [32], due to the excellent thermal stability (melting point 2780~2920 K, thermal expansion 5.8 × 10^−6^/°C). Therefore, HfO_2_-based metasurfaces are suitable for high-power applications. In recent years, HfO_2_ has been exploited in constructing metasurfaces [33,34,35]. However, these meta-lenses are characterized by a narrow FOV [33]. On the other hand, The high-power systems usually exploit the 1.319 µm laser as the source [36]. Thus, HfO_2_ metasurfaces made of refractory materials HfO_2_ with a large field of view at infrared wavelengths is very desired for high-power optical systems, which can be employed to direct or collect high-power light beams with a large FOV. The applications include staring imaging laser radars, light projection, etc.

In this study, novel HfO_2_ quadratic meta-reflectors are investigated with a large FOV at infrared wavelengths. The meta-reflectors consist of nanopillars with a quadratic phase profile atop a distributed Bragg reflector (DBR)/Au-mirror bottom. First, the general principles of designing infrared HfO_2_ metasurfaces are demonstrated by investigating the geometrical dependence of phase difference. Then, two meta-reflectors are constructed following a quadratic phase profile, and their optical properties of the FOV, resolution, and focal depth are researched when focusing light beam. Furthermore, the angle range is also analyzed when the meta-reflectors deflect a Gaussian point source on the focal plane. To our knowledge, the investigation is not reported in the previous literature.

## 2. Design Principles and Methods

### 2.1. Quadratic Phase Profile

The phase profile of the studied meta-reflectors is a quadratic function of the radial distance *r* to the reflector center. Therefore, the meta-reflectors are termed “quadratic meta-reflector”:

(1) $$\varphi \left(r\right)=k_{0}\frac{r^{2}}{2f}$$

where *k*_0_ = 2*π*/*λ* is the vacuum wavenumber, *f* is the focal distance, and r∈[0, f]. Here, the *x*-*y* plane is defined as the plane of the meta-reflectors, and the origin of coordinates is located at the reflector center. Therefore, *r*^2^ = *x*^2^ + *y*^2^. If the collimated light has an incident angle *θ* in the *x*-*z* plane, the wavefront phase of the reflected light can be expressed as:

(2) $$\varphi \left(r\right)=k_{0}\frac{r^{2}}{2f}+k_{0}x\sin\theta =\frac{k_{0}}{2f}\left[{\left(x+f\sin\theta \right)}^{2}+y^{2}\right]-\frac{fk_{0}\sin^{2}\theta }{2}$$

**Figure 1:**
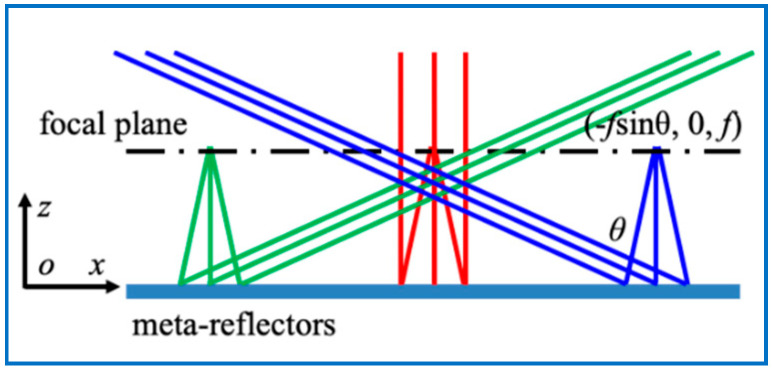
Quadratic meta-reflectors. The colorful lines represent the light beams with different incident angles *θ* and the focal spots at the positions with coordinates (−*f*sin*θ*, 0, *f*).

As shown in Figure 1, it is obvious that the light beams with different incident angle *θ* are focused on the focal plane with coordinates (−*f*sin*θ*, 0, *f*). If a Gaussian source is localized on the focal plane with coordinates (−*f*sin*θ*, 0, *f*), light could be reflected as a collimated light with oblique angle *θ*. The relationship between the oblique angle *θ* and the *x*-coordinate $\Delta_{x}$ of the focal position is that $\Delta_{x}=-f\sin\theta$. From Equation (2), it is known that the oblique angle *θ* can be 90° theoretically when $\Delta_{x}=-f$. The large FOV is achieved by the quadratic phase profile *φ*(*r*) = *k*_0_*r*^2^/(2*f*) because it has a high phase gradient (d*φ*/d*r* = *k*_0_*r*/*f*, d*φ*/d*r* = *k*_0_ when *r* = *f*) to deflect the beam. To realize the high phase gradient, the lattice constant *P* should be small, when the phase difference is limited by the maximum value 2π. With a small lattice constant, the radius of nanopillars should be small too, leading to a high aspect ratio. Thus, the nanostructures should have high dielectric coefficients, which can achieve the entire 2π phase coverage with the smaller radius-change range [37], avoiding a high aspect ratio.

### 2.2. Schematic of Nanopillars

**Figure 2:**
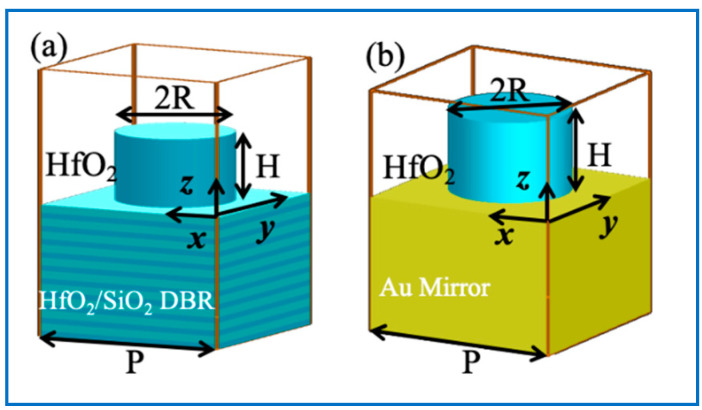
Schematics of the unit cells constituting meta-reflectors: (**a**) 3D view of HfO_2_ nanopillars with DBR as a bottom mirror that is made of alternately arranged 159-nm-thick HfO_2_ layers and 228-nm-thick SiO_2_ layers; (**b**) 3D view of HfO_2_ nanopillars with Au film as a bottom mirror. The lattice constant is *P* = 0.66 μm.

Metasurfaces are composed of arrayed nanostructures with different sizes and arrangements. Two configurations of the unit cells constituting HfO_2_ meta-reflectors are presented in Figure 2. The unit cells contain a HfO_2_ nanopillar with radius R and Height H and a DBR (in Figure 2a) or an Au film (in Figure 2b) as a bottom mirror. 1.319 µm is set as the working wavelength *λ* because it is always employed in high-power systems with Nd: YAG (neodymium-doped yttrium aluminium garnet; Nd:Y_3_Al_5_O_12_) lasers [36]. The DBR is made of ten alternately arranged 159-nm-thick HfO_2_ layers and ten 228-nm-thick SiO_2_ layers, where 159 nm and 228 nm are the product of *n*_HfO2/SiO2_ and *λ*/4. The high reflectance comes from the constructive interference between the incidence and the reflected light in every layer with optical thickness *n*_HfO2/SiO2_*λ*/4. The incidence is assumed to be a plane wave, propagating along the *z*-axis direction and being polarized along the *x*-axis direction. The incident light is reflected by the DBR/Au bottom mirror and therefore it passed through the nanopillars two times. The HfO_2_ nanopillars are cylinder and isotropic for polarization, so the metasurfaces are insensitive to polarization.

According to the Nyquist theorem when designing metasurfaces [38], the lattice constant *P* should satisfy the required sampling criterion *P* ≤ *λ*/(2*NA*), where *NA* = *n*sin*α*, *n* is the refractive index of air, and *α* is the field angle (deflecting angle of light). The meta-reflectors are designed for a large FOV, so *α* is set as *α* = 90°. Therefore, *P* ≤ *λ*/(2*NA*) = *λ*/(2·1·sin90°) = *λ*/2 = 660 nm. On the other hand, a small value of *P* could lead to a high aspect ratio of HfO_2_ nanopillars. Here, the value of *P* is chosen as the maximum value *P* = 660 nm. The high dielectric coefficient of HfO_2_ is important to realize the entire 2π phase coverage with the smaller geometrical change of nanostructures [37], which can avoid high aspect ratios. On the other hand, the electric field can be confined in the nanopillars with high dielectric coefficients, voiding the electric crosstalk between different nanopillars in the meta-reflectors. With different geometrical parameters *H* and *R*, the phase difference of reflected light can be tuned. Once the phase-difference tuning covers the 0–2π range, the quadratic phase profile can be realized by the unit cells of HfO_2_ nanopillars.

### 2.3. Methods

All the simulations in this study are implemented by the 3-dimensional finite-difference time-domain (3D FDTD) algorithm with the commercial software package FDTD solutions provided by Lumerical Solutions, Inc. in Vancouver, BC, Canada. The simulation region was surrounded with periodic boundaries in the *x*-axis and *y*-axis directions and perfectly matched layers in the *z*-axis direction. The dielectric function data of hafnium oxide (HfO_2_) refers to the experimental data [39]. All the other optical constants were taken directly from the database of the FDTD software.

## 3. Results and Discussions

### 3.1. Geometrical Dependence of Reflection

**Figure 3:**
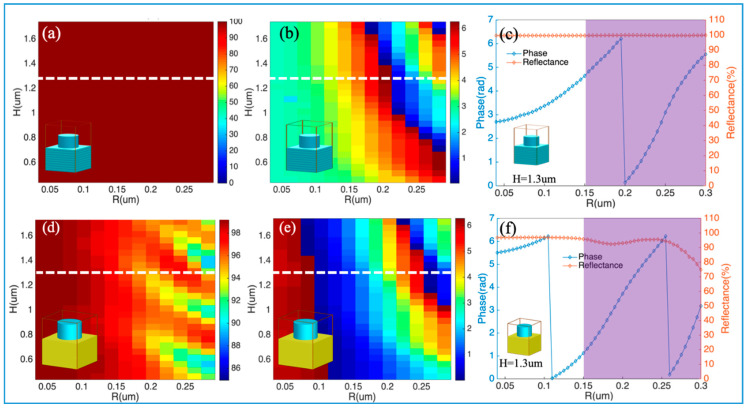
Geometrical dependence of reflection on parameters *R* and *H*: (**a**) reflectance and (**b**) phase difference for light reflected by a DBR reflector with HfO_2_ nanopillars atop; (**c**) reflectance and phase difference with constant height *H* = 1.3 μm, marked by a white dotted line in (**a**) and (**b**); (**d**) reflectance and (**e**) phase difference for light reflected by an Au film with HfO_2_ nanopillars atop. (**f**) reflectance and phase difference with Au film as a bottom mirror and constant height *H* = 1.3 μm, marked by a white dotted line in (**d**) and (**e**). The purple shaded areas of (**c**) and (**f**) are in the *R* range from 0.15 µm to 0.3 µm, in which the phase difference covers the 0–2π range with high reflectance. The inserted images show the unit cells with DBR or Au Film as a bottom mirror.

**Figure A1:**
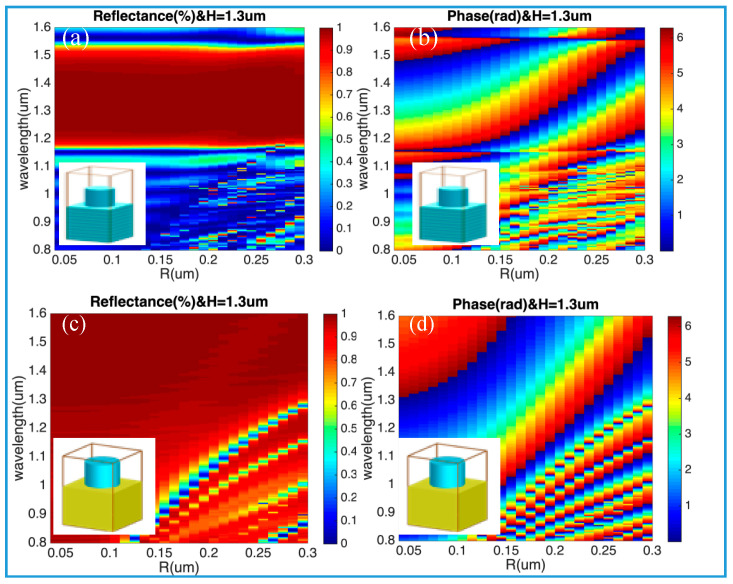
Geometrical dependence of reflection on parameters *R* and *λ*: (**a**) reflectance and (**b**) phase difference for light reflected by a DBR reflector with HfO_2_ nanopillars atop; (**c**) reflectance and (**d**) phase difference for light reflected by an Au film with HfO_2_ nanopillars atop. Here, the height is constant H = 1.3 μm. The inserted images show the schematics of the unit cells.

To demonstrate the choice of the unit cells for designing HfO_2_ meta-reflectors, the geometrical dependence of HfO_2_ nanopillars’ phase difference is analyzed via changing the parameters *R* and *H*. Reflectance and phase difference of the normally incident light, which is reflected by a DBR mirror with HfO_2_ nanopillars atop, are shown in Figure 3a,b. The inserted images show the schematics of the unit cells. The incident light is reflected almost totally by the DBR bottom mirror (the reflectance is almost 100%) and the phase difference covers the 0–2π range. With constant height *H* = 1.3 μm, the reflectance and phase difference are shown in Figure 3c, which is marked by a white dotted line in Figure 3a,b. When radius *R* is the only variable, the phase difference can still cover the 0–2π range with high reflectance. Similarly, with Au film as a bottom mirror, the reflectance and phase difference are shown in Figure 3d–f. The inserted images show the schematics of the unit cells. The geometrical dependence of reflection on parameters *R* and wavelength *λ* could be found in Figure A1 of Appendix A.

In the purple shaded areas of Figure 3c,f, the phase difference covers the 0–2π range with high reflectance. In the areas, the unit cells with 16 phase levels are chosen to design the quadratic meta-reflectors and the radii of nanopillars are 150 nm, 160 nm, 300 nm by step of 10 nm. The aspect ratios *H*/(2*R*) are between 2.17 and 4.33. In Ref. [33], an ultraviolet metalens was constructed and fabricated, which made of high-aspect-ratio HfO_2_ circular pillars of varying radii. Moreover, the highest aspect ratio of fabricated HfO_2_ nanostructures can be 13 [40]. Thus, the aspect ratio of HfO_2_ nanopillars in the study could be feasible for fabrication.

### 3.2. Large FOV of Light Focusing

**Figure 4:**
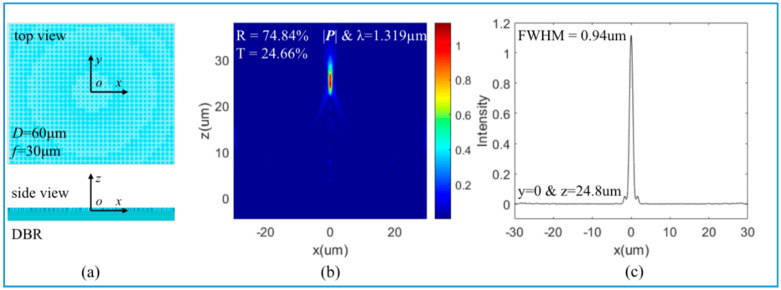
Focal features of meta-reflectors with DBR: (**a**) schematic of a meta-reflector with DBR as bottom mirror, size *D* = 60 μm and focal distance *f* = 30 μm; (**b**) Focal profile of the reflected light on the *x*-*z* plane with *y* = 0; (**c**) FWHM of the focus spot at *z* = 24.8 μm.

**Figure 5:**
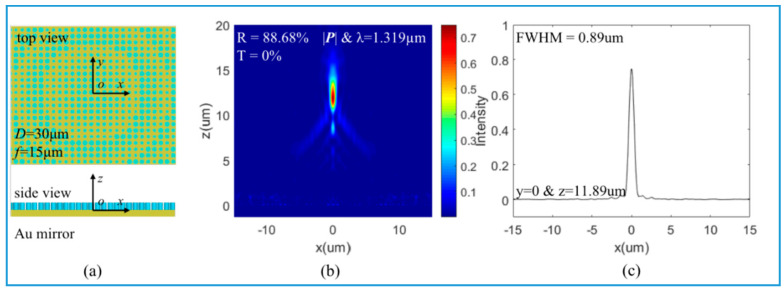
Focal features of meta-reflectors with Au film: (**a**) schematic of a meta-reflector with Au film as a bottom mirror, size *D* = 30 μm and focal distance *f* = 15 μm; (**b**) Focal profile of the reflected light on the *x*-*z* plane with y = 0; (**c**) FWHM of the focus spot at *z* = 11.89 μm.

With DBR as a bottom mirror, a quadratic meta-reflector with aperture *D* = 60 μm and focal distance *f* = 30 μm is constructed, as shown in Figure 4a, which is based on the 16 phase levels of the blue curve in Figure 3c. The incident light is x-polarized and propagates in the *z*-axis direction. The reflected light is focused at the focal plane’s center, as shown in Figure 4b. The reflectance of the meta-reflector is 74.84%, lower than the reflectance 100% of the nanopillars in Figure 3c. This is because the DBR substrate mirror has a strong dependence on the incidence direction. In the study, the DBR reflectance is optimal only in the normal direction. After a normally incident plane wave passes through the meta-reflector with phase gradient *k*_r_ = *d*φ(*r*)/*dr* = *k*_0_*r*/*f*, its propagation direction becomes oblique, leading to a transmission 24.66%. In Figure 4b, the meta-reflector has a long focal depth, more than 5 µm. In Figure 4c, the full width at half maximum (FWHM) of the focal spot is 0.94 µm, which means the subwavelength resolution. When the bottom mirror is an Au film, a quadratic meta-reflector with aperture *D* = 30 μm and focal distance *f* = 15 μm is constructed based on the 16 phase levels of the blue curve in Figure 3f. The light-focusing results are shown in Figure 5. The reflectance is 88.68% while the transmittance is zero. With Au film as a bottom mirror, 11.32% of energy is dissipated due to ohmic loss. It also has a long focal depth and a subwavelength FWHM of the focal spot. Here, the two kinds of meta-reflectors have the same NA, so the resolutions (FWHM) should be similar, 0.94 µm and 0.89 µm. Moreover, the oblique propagation direction in DBR also could introduce phase error, leading to a little wider FWHM.

**Figure 6:**
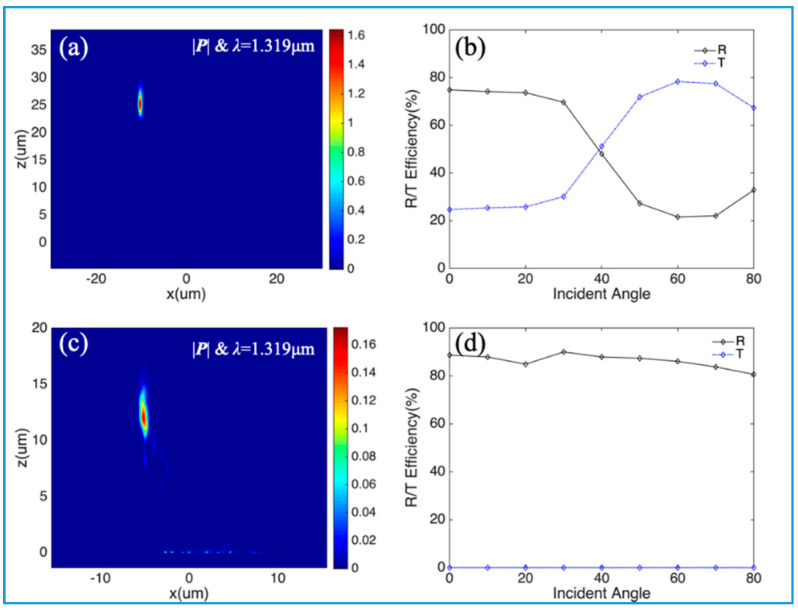
Large-FOV focusing of meta-reflectors: (**a**) Focal profile of light with an incident angle 20°, which is reflected by the meta-reflector with DBR as a bottom mirror in Figure 4a; (**b**) Reflectance and Transmittance of light with different incident angles from 0° to 80°, which is reflected by the meta-reflector in (**a**); (**c**) Focal profile of light with an incident angle 20°, which is reflected by the meta-reflector with Au film as a bottom mirror in Figure 5a; (**d**) Reflectance and Transmittance of light with different incident angles from 0° to 80°, which is reflected by the meta-reflector in (**c**).

**Figure A2:**
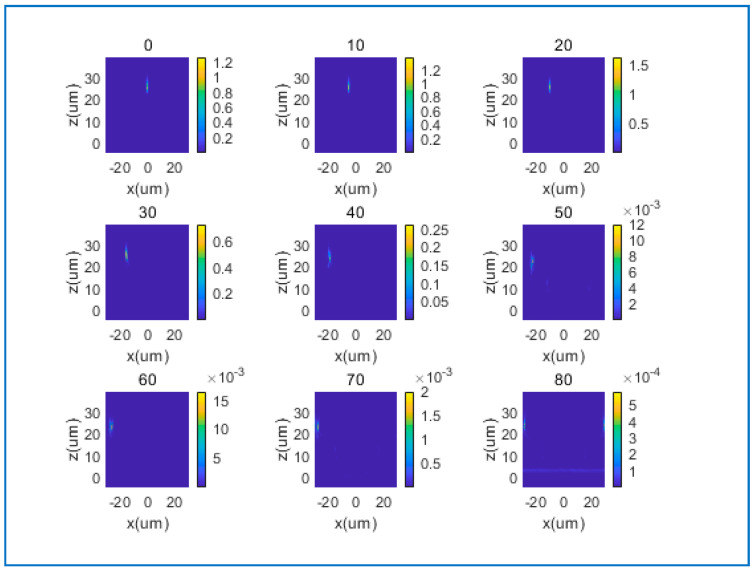
Focal profile of light with incident angles from 0° to 80°, which is reflected by the meta-reflector with DBR as bottom mirror in Figure 4a.

**Figure A3:**
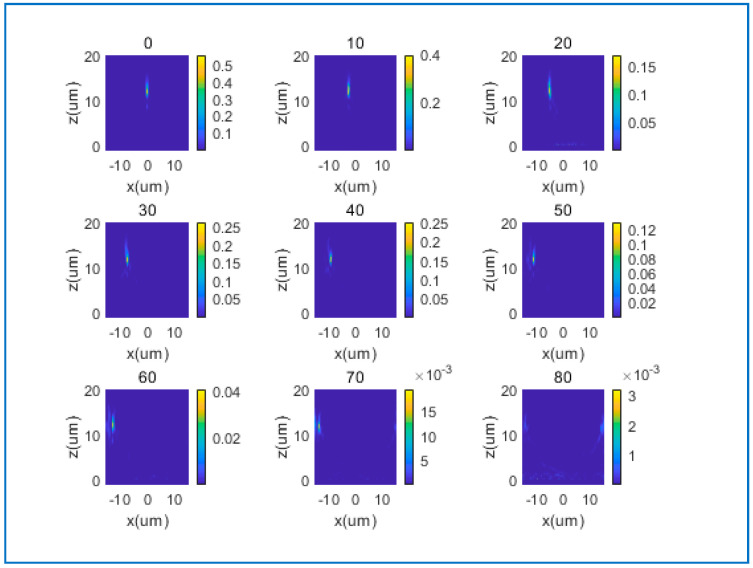
Focal profile of light with incident angles from 0° to 80°, which is reflected by the meta-reflector with Au film as bottom mirror in Figure 5a.

When the collimated light has an incident angle *θ* in the *x*-*z* plane in Figure 4a and Figure 5a, the focal spots will still stay on the focal plane but have a position offset, as shown in Figure 6. With an incident angle of 20°, the light is focused on the focal plane with a position offset $\Delta_{x}=-\sin\theta f$, as shown in Figure 6a,c. With DBR as a bottom mirror, the reflectance decreases (the transmittance increases) when the incident angle increases from 0° to 80°, due to a bigger oblique angle, as shown in Figure 6b. In the case of the meta-reflector with Au film as a bottom mirror, the reflectance always stays above 80% and the transmittance is zero, as shown in Figure 6d. The focal profiles of light with different incident angles could be found in Figure A2 and Figure A3 of Appendix A.

### 3.3. Large FOV of Light Deflecting

**Figure 7:**
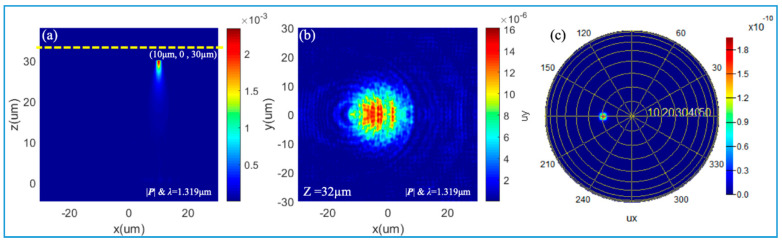
Deflecting features of meta-reflectors: (**a**) A Gaussian source is localized on the focal plane with coordinates (10 μm, 0, 30 μm), which is reflected by the meta-reflector with DBR in Figure 4a; (**b**) Intensity distribution on the x-y plane, marked by a yellow dot line in (**a**); (**c**) Intensity distribution of the reflected light in the coordinate system of inclination angle and azimuth angle.

Besides the wide-angle focusing properties, the quadratic meta-reflectors also have a large-FOV feature when deflecting a beam. As shown in Figure 7a, a Gaussian point source is localized on the focal plane with coordinates (10 μm, 0, 30 μm), i.e., $\Delta_{x}$ = 10 µm. Here, the HfO_2_ nanopillars are located on a DBR bottom mirror. The intensity distribution of the reflected light on the *x*-*y* plane, marked by a yellow dot line, is shown in Figure 7b. The far-field radiation direction of the reflected light can be calculated by the equation $\theta =-\arcsin\left(\Delta_{x}/f\right)=19.47{}^{\circ}$. It is verified by the intensity distribution of the reflected light in the coordinate system of the inclination angle and azimuth angle in Figure 7c.

**Figure 8:**
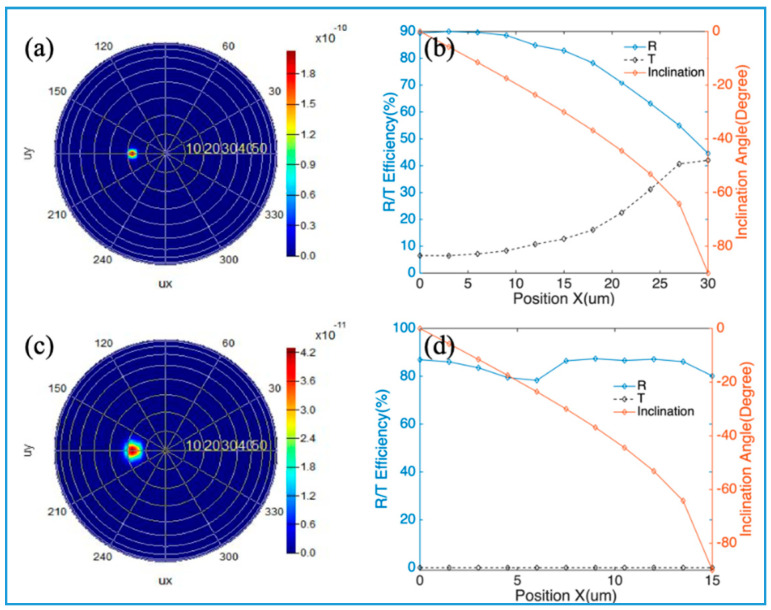
Large-FOV deflecting of meta-reflectors: (**a**) Intensity distribution of the reflected light in the coordinate system of inclination angle and azimuth angle. The Gaussian source is localized on the focal plane with coordinates (9 μm, 0, 30 μm), which is reflected by the meta-reflector with DBR in Figure 4a; (**b**) Reflectance, transmittance and inclination angle of the reflected light as a function of the *x*-coordinate of source on the focal plane of the meta-reflector with DBR; (**c**) Intensity distribution of the reflected light in the coordinate system of inclination angle and azimuth angle. The source is localized on the focal plane with coordinates (4.5 μm, 0, 15 μm), which is reflected by the meta-reflector with Au film in Figure 5a; (**d**) Reflectance, transmittance, and inclination angle of the reflected light as a function of the *x*-coordinate of source on the focal plane of the meta-reflector with Au film.

**Figure A4:**
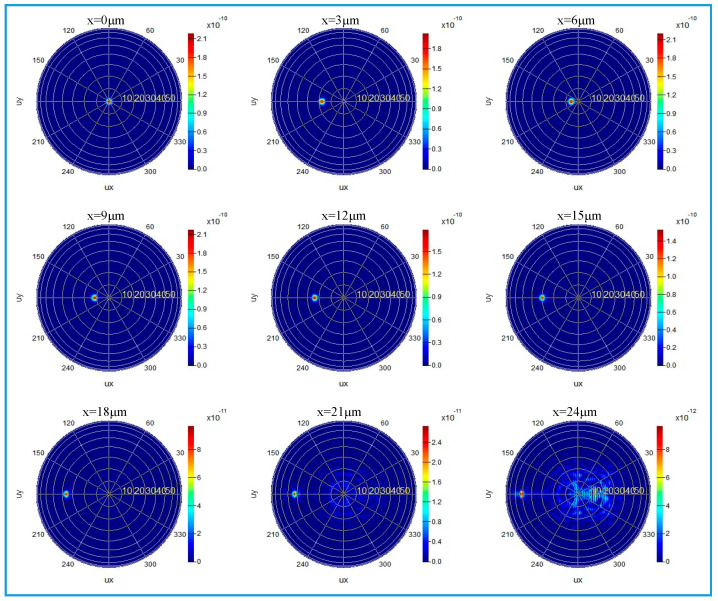
Intensity distribution of the reflected light in the coordinate system of inclination angle and azimuth angle. The Gaussian source is localized on the focal plane with coordinates (0–24 μm, 0, 30 μm), which is reflected by the meta-reflector with DBR in Figure 4a.

**Figure A5:**
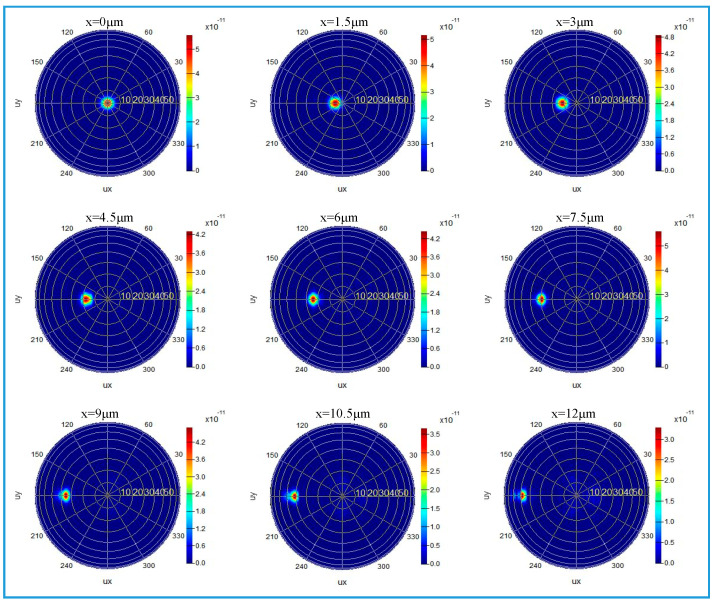
Intensity distribution of the reflected light in the coordinate system of inclination angle and azimuth angle. The Gaussian source is localized on the focal plane with coordinates (0–12 μm, 0, 15 μm), which is reflected by the meta-reflector with Au film in Figure 5a.

By changing the horizontal position between the point source and the meta-reflectors’ center, the radiation direction of reflected beams can be readily tuned and deflected in a large FOV, as shown in Figure 8. As it is similar to the focusing case in Figure 6, the meta-reflector with DBR has a lower reflectance when the source has a bigger *x*-coordinate (therefore a bigger oblique angle), as shown in Figure 8a,b. If the bottom mirror is an Au film, the reflectance is always above 80%, as shown in Figure 8c,d. The brown lines show the inclination angles of the reflected light when the source’s *x*-coordinate changes. The FOV is bigger than 80°. When the source has different *x*-coordinates, the intensity distributions of the reflected light in the coordinate system of inclination angle and azimuth angle could be found in Figure A4 and Figure A5 of Appendix A. By changing the horizontal position of the point source on the focal plane of the meta-reflectors, the radiation direction of output beams can be tuned and deflected in a large FOV. They could be employed in many applications such as laser radar and optical projectors, where the meta-reflectors can deflect the output beam of the point source on the focal plane to different radiation directions according to different horizontal positions.

In the study, the two substrates, Au films and HfO_2_-SiO_2_ DBRs are chosen because they are the very typical mirrors based on metal (non-dielectric) and dielectric materials. The DBRs have a small ohmic loss and a high damage threshold. However, DBR has a dependence on the angle, leading to low efficiency when the light is deflected with a large angle. The Au ones have no angle dependence and high efficiency at a large FOV. However, the Au mirror has ohmic losses, and Au films are employed in the high-power case where the power density is below the maximum value [41]. Thus, the DBR-based meta-reflectors can be employed in the applications with a very high power density and at a constant incident/projection angle, while the Au ones can be employed in high-power systems with a smaller power density and at a changing incident/projection angle. Compared to other meta-lenses which focus light with different incident angles on the same focal point, the studied meta-reflectors focus the light with different incident angles on the different focal points on the same plane. It means that the studied meta-reflectors can transform the rotational symmetry associated with the off-axis incident light to the translational symmetry. Thus, they can be employed as lenses of staring imaging laser radars. Moreover, they can be employed to project laser beams. A large FOV will be beneficial to direct a high-power impulse into the desirable direction, just by changing the horizontal position of the point source on the focal plane of the meta-reflectors.

## 4. Conclusions

In summary, we have demonstrated quadratic meta-reflectors in the near-infrared band, which are made of HfO_2_ nanopillars of varying radiuses. The nanopillars are designed to obtain 2π phase control at infrared wavelength 1.319 µm. The design principles of HfO_2_ nanopillars are demonstrated for the HfO_2_-metasurface applications. We have designed two quadratic meta-reflectors with DBR or Au film as a bottom mirror, which show the compound-eye feature, subwavelength resolution, and long focal depth. Furthermore, the two quadratic reflectors show a large FOV with an angle range of approximately ±80° when focusing/deflecting a beam. When focusing light, the meta-reflector with Au film always has a reflectance of about 80% in its view and the reflectance of the DBR one is 20–80% (smaller at a larger angle). When deflecting light, the reflectance with Au film is still about 80% in all view and that of the DBR one is 40–90%. This research on quadratic meta-reflectors paves the way for a flat optical metamaterial with a quadratic large FOV for imaging and deflecting, which have the potentials to simplify the optical–mechanical complexity of high-power infrared systems such as laser radars and projectors.
